# Experiences and self-care of pregnant nurses with gestational diabetes mellitus: a qualitative study

**DOI:** 10.1186/s12912-023-01679-x

**Published:** 2024-01-11

**Authors:** Jing He, Hui Wang, Xiaoli Chen

**Affiliations:** 1 Nursing department, Tongji Hospital of Tongji Medical College of Huazhong, University of Science and Technology, NO.1095 Jiefang Avenue, Wuhan, China; 2 School of Nursing, Tongji Medical College of Huazhong, University of Science and Technology, NO.13 Aviation Road, Wuhan, China; 3 School of Nursing, Wuhan University, NO. 115 Donghu Road, Wuhan, China

**Keywords:** Gestational diabetes mellitus, Qualitative research, Nurses, Disease experiences, Self-care

## Abstract

**Background:**

Pregnant nurses are at high risk of developing gestational diabetes mellitus (GDM), and nurses diagnosed with GDM face challenges in balancing disease management and work, which affects maternal and child health and the quality of care. GDM requires significant changes to lifestyle and physical activity to control blood glucose levels, which is key to reducing adverse pregnancy outcomes. However, few studies have focused on the experiences of pregnant nurses with GDM. This study aimed to gain insight into the experiences of pregnant nurses with GDM in China in terms of their illness, work burdens, and self-care.

**Methods:**

This qualitative study used an interpretative phenomenological analysis. Face-to-face semi-structured in-depth interviews were conducted with pregnant nurses with GDM to investigate their experiences and self-care. The study was performed at Chongqing’s maternal and child health hospital in China. A purposive sampling was used. Nine pregnant nurses diagnosed with GDM were recruited and interviewed.

**Results:**

The interview data generated four themes and 11 sub-themes. The four themes were ‘the perceptions and feelings of GDM’, ‘experiences of lifestyle changes’, ‘social support needs’, and ‘health expectations and risk perception.’

**Conclusion:**

Many factors such as the unique occupational environment, overwork, occupational pressure, shift work, family status, and education level may lead to difficulties in managing blood glucose in nurses with GDM. These findings suggest that managers should pay more attention to nurses with GDM and develop personalized medical care and work arrangements. These measures can improve the self-care and well-being of nurses with GDM and promote the health of nurses and their offspring.

**Supplementary Information:**

The online version contains supplementary material available at 10.1186/s12912-023-01679-x.

## Introduction

According to Chinese health statistics, there were approximately 5.02 million registered nursing staff in the country in 2021. Women accounted for 96.7% of these, and 85% of female nurses were of childbearing age between the ages of 18 to 44 years [1]. There were 8.47 billion visits to medical and health institutions in China in 2021 while the proportion of registered nurses per 1,000 population was 3.56, indicating a high demand on nursing teams and nursing service quality [1]. Shen et al. observed that the status quo of nurses’ clinical work represented continuous busyness with regular overtime involving shift relief and the writing up of nursing records and quality control information) [2]. Occupational challenges, as well as the physical and mental changes occurring during pregnancy, make it difficult for nurses to maintain a healthy lifestyle; these challenges include long working hours, night work, stress, and working in a COVID-19 background, amongst others [3, 4].

Studies have shown that specific aspects of nursing work, such as long shifts, can worsen insulin resistance during pregnancy [5]. Shan et al. and Pan et al. found that night shifts and job-related psychosocial stress were independent risk factors for type 2 diabetes mellitus (T2DM) in nurses [6, 7]. In addition, sleep disturbances and stress may lead to insulin resistance, impaired glucose regulation, and the development of T2DM [8, 9]. The pathological mechanism of gestational diabetes mellitus (GDM) is similar to that of T2DM and is associated with insulin resistance and islet cell dysfunction [10]. The results of these studies suggest that pregnant nurses are likely to be at high risk of developing GDM; however, there is little information on the occurrence of GDM in nurses.

Once a pregnant nurse is diagnosed with GDM, daily work tasks and blood glucose management during pregnancy become more challenging. These complex scenarios during pregnancy can exacerbate burnout and job dissatisfaction, as well as limiting effective glycemic management and self-care during pregnancy [4, 11]. Weschenfelder et al. found that poor sleep status in women with GDM was independently related to the need to use long-acting insulin at night [12]. Circadian disruption in shift work can impair blood glucose regulation [13], and a study from China found that reduced durations of nighttime sleep were associated with poor blood sugar control in women with GDM [14]. Moreover, GDM increases the risk of short-term adverse perinatal outcomes and long-term metabolic diseases [15, 16].

Guidelines from the American Diabetes Association (ADA) recommend that adopting a healthy lifestyle during GDM significantly reduces the risk of adverse outcomes, indicating the importance of blood sugar control and management [17]. Unfortunately, although nurses with GDM may understand the importance of such requirements, they may not be able to obtain regular exercise, adequate stress management sleep, or eat a healthy diet [18]. Nurses may also consider it too difficult to balance busy work schedules with regular self-care behaviors during pregnancy [4]. In addition, nurses with GDM need to practice proper blood glucose management and the self-care of a healthy lifestyle during pregnancy to ensure the health and well-being of both themselves and the fetus [4]. However, there are no current studies addressing the work experience, blood glucose management, and self-care of pregnant nurses with GDM.

It is thus important to investigate the experiences of nurses with GDM to determine the problems they face and their ability to manage both the disease and self-care during pregnancy to identify and mitigate the possible risks and problems in this field. This study aimed to investigate and analyze the experiences of pregnant nurses with GDM. The findings can provide a comprehensive understanding of the needs and difficulties of nurses with GDM, help to develop personalized nursing management plans, reduce the risk of metabolic diseases caused by GDM, and promote the health of nurses and their offspring.

## Methods

### Study design

This study was undertaken as an interpretative phenomenological analysis (IPA) of qualitative research to explore the experiences and feelings of pregnant nurses with GDM. The purpose of an IPA is to explore how participants understand their own personal and social worlds [19]. This method can be used to elaborate on the interviewee’s personal views and understanding of the object or events in a specific life experience. Our view of the world consists of the interaction between the raw material of the world and the complex mental framework developed by personal backgrounds and life experiences [20]. This interaction helps us construct unique interpretations of the world. Researchers need to identify how participants try to make sense of their personal and social worlds in a particular cultural context. The researcher (JH) who conducted the interviews was a female nursing postgraduate student and qualified Psychological Consultant with expert psychological knowledge and interview experience.

This study was approved by the Ethics Committee of the Chongqing Health Center for Women and Children (Number: 2020-022), and participants provided signed informed consent to participate in the study before taking part. The Consolidated Criteria for Reporting Qualitative Studies (COREQ) checklist was used in the study [21].

### Sampling and participants

The study participants were selected using the purposive sampling approach combined with maximum variation (in terms of age, parity, pre-pregnancy body mass index, educational background, and clinical department) [22]. The pregnant nurses with GDM were interviewed and information was collected. The study used Information Power to determine the sample size; this includes four specifications, namely, a narrow study aim, dense sample specificity, strong dialogue quality, and case strategy [23]. The obstetrics GDM specialist outpatient nurse was responsible for recruiting participants who met the criteria at Chongqing Health Center for Women and Children. One researcher (JH) was responsible for contacting and identifying participants willing to participate in the study and making a telephone appointment to be interviewed at the next outpatient obstetric visit after the diagnosis of GDM.

The inclusion criteria were as follows; pregnant nurses with professional qualifications, and patients using the 75-gram Oral Glucose Tolerance Test (OGTT) who met the diagnostic criteria of the International Association of Diabetes in Pregnancy Study Group (IADPSG). Any abnormalities in these indicators were diagnosed as GDM. The exclusion criteria were patients with other pregnancy complications or other underlying medical conditions.

### Data collection

The data were collected through personal face-to-face semi-structured interviews using the Chinese language. Before the research interview, we introduced the purpose, significance, methods and content of the research to the participants and established a familiar relationship. Written informed consent was obtained from the participants for the recording and taking of notes. The collection site was a quiet office with only one participant and one researcher (JH) in the clinic. The subjects were anonymized with letters and numbers (e.g., G1 = Participant 1).

A total of 20 eligible participants were recruited and 11 nurses declined to participate. Nine pregnant nurses did not have time for the interview and two pregnant nurses did not want to talk about GDM. Each participant was interviewed only once. The interviews were completed from May to October 2020 and lasted on average 41.2 min. The nurses were asked to describe their experiences and feelings after being diagnosed with GDM. The interview questions are shown in Table 1. All information relating to the interview was kept on secure computers and password protected by two researchers (JH and XC). One researcher translated the Chinese quotations into English while another, a Chinese person living in an English-speaking country, translated them back to ensure that the original meaning was preserved.

**Table 1:** Semi-structured interview questions

No.	Interview questions
1	How did you feel when you learned you had been diagnosed with GDM? What do you think of GDM?
2	How did your work and life change and challenge when GDM existed?
3	Have you encountered any difficulties during blood glucose management in GDM? What kind of help do you want most?
4	As a caregiver who cares for others, how do you manage blood glucose to care for yourself?
5	What are your prospects for the future health of yourself and your baby?
6	The above questions taught us about your experience dealing with GDM illness, workload, and self-care. Is there anything else you need to add?

### Data analysis

The analysis followed the procedures outlined by the IPA [24]. The specific steps of analysis were: (1) Repeated reading of the transcribed text; (2) Analysis of each code line by line, looking for descriptive, linguistic, and conceptual points of interest, and forming preliminary comments and analysis; (3) Transformation of the initial notes into emerging emotional themes that capture the core of the participants’ experience, leading to the proposal of themes; (4) Cases are collected and converted into a separate topic table, and correlations between the topics are identified; (5) Analysis of subsequent cases, grouping and converting each case into a separate topic table; (6) Comparison of the topics by searching for convergence and divergence, that is, looking for thematic patterns between individual cases. The IPA endorses a two-stage interpretive process, implying that researchers are required to construct meaning by understanding participants [24].

NVivo12 (QSR International Pty Ltd. Version 10, 2014) was used in the management, shaping, and analysis of the text of qualitative data. The results of data transcription and analysis were returned to the interview subject for confirmation and questions or ambiguities were clarified. Two researchers (JH and XC) acquired, analyzed, summarized, and supplemented the recording materials and field notes within 24 h. The recorded material was transcribed verbatim into text. The participants listened to the recorded material, compared the transcribed material, and added and recorded nonverbal information. Three researchers (JH, HW, and XC) were involved in the coding of the data. HW and XC are nursing professors working in a hospital and university, respectively, with rich experience in nursing management and obstetric care. Different analyses by different researchers could potentially extract different elements from the participants’ accounts. Therefore, a collaborative approach to analysis ensures the comprehensiveness and credibility of the final report [25].

## Results

### Participant characteristics

Nine pregnant nurses that had been diagnosed with GDM were interviewed. The participants were between 25 and 33 years old with an average age of 29 years. The interviewees were included five primiparas and four multiparas with pre-pregnancy body mass index (BMI) of 19.5–28.9 kg/m^2^. Three pregnant nurses were educated to junior college level and six nurses were educated to undergraduate level. The characteristics of the participants are summarized in Table 2.

In-depth interviews were conducted on the experiences and self-care of nine pregnant nurses diagnosed with GDM, and the interview data comprising 571 nodes were analysed and coded. In addition, the interview data formed 11 sub-themes and four themes. The findings were presented as four core themes: the perception and feelings of GDM, experiences of lifestyle changes, social support needs, and health expectations and risk perception (Table 3).

**Table 2:** Characteristics of pregnant nurses with gestational diabetes mellitus (n = 9)

No.	Gravidity	Parity	Height/cm	Pre-BMI/ kg/m^2^	Fasting	1-h	2-h	Education	Professional title
G1	1	0	160	19.5	5.3	6.3	7.8	Junior college	No
G2	3	1	155	22.5	4.8	10.4	9.4	Bachelor	Senior nurse
G3	3	1	155	23.7	5.2	8.9	8.2	Junior college	Senior nurse
G4	5	0	158	24.0	4.9	10.5	7.7	Bachelor	Supervisor nurse
G5	3	0	155	22.1	4.3	7.9	8.7	Bachelor	No
G6	3	1	160	28.9	4.7	12.2	7.9	Bachelor	Senior nurse
G7	2	0	161	22.4	5.8	11.8	8.2	Junior college	No
G8	2	1	162	21.0	4.4	11.7	8.2	Bachelor	Senior nurse
G9	2	0	163	21.8	4.1	10.9	10.1	Bachelor	Senior nurse

Fasting, fasting plasma glucose of oral glucose tolerance test (OGTT); 1-h, OGTT 1-h plasma glucose; 2-h, OGTT 2-h plasma glucose (mmol/L); Pre-BMI, Pre-pregnant body mass index.

**Table 3:** Four themes and 11 sub-themes

Themes	Sub-themes
The perceptions and feelings of GDM	Emotional responses to the diagnosis
	Self-reflection
	Attitudes and perceptions of GDM
Experiences of lifestyle changes	Conflict between diet management and clinical work
	Fatigue from physical labor
	Blood glucose monitoring and emotional shock
Social support needs	Humanistic care needs
	Family support needs
	Available professional support
Health expectations and risk perception	The responsibilities and expectations of motherhood
	Awareness of self-health and image

GDM, Gestational diabetes mellitus

### The perceptions and feelings of GDM

The first topic of the study was the perception and feelings associated with GDM. Pregnant nurses reported emotional shock at the diagnosis of GDM, which caused them to question their health status. Pregnant nurses with GDM immediately reflected on the influence of diet and exercise on their blood glucose levels based on their medical knowledge. In addition, nurses also expressed perceptions of GDM, including issues such as controllability and disease stigma. Three sub-themes were identified in relation to this topic.

#### Emotional response to the diagnosis

Pregnant nurses with GDM were interviewed for this study. The pregnant nurses with GDM were psychologically shocked, amazed, lost, and sad when they became aware of their blood glucose results. However, as nurses, as they had medical knowledge and understood that GDM was controllable, these adverse emotional reactions were rapidly alleviated. At this stage, the nurses considered that excessive anxiety over the controllability of blood glucose was unnecessary and that maintaining a good state of mind was important for fetal health. In addition, some nurses said a diagnosis of GDM was a “wake-up call” to past unhealthy eating habits. Typical statements conveying their emotions are shown below.

*“My OGTT results were only 0.2 (mmol/l) above the diagnostic criteria 2-h plasma glucose. However, the doctor said it was GDM, although I was reluctant to accept the result… (sighs).” (G1)*.

*“When I got the OGTT results, I was so upset. I couldn’t control my emotions and cried for a while. I began to resist anything sweet and dared not eat fruit.” (G5)*.

#### Self-reflection

Pregnant nurses reported self-reflection on the possible causes of the abnormal OGTT results immediately after the diagnosis, reflecting their professional knowledge and understanding of health. They considered the risk factors for GDM in a medical context, including overindulgence in fruit and sweets, drinking soft drinks, insufficient exercise, obesity, and eating takeaways.

*“I know that my pre-pregnancy weight (overweight) and PCOS (polycystic ovary syndrome) are high-risk factors for GDM. I need to stay in a good mood and control my diet, including limiting anything too sweet or salty.” (G3)*.

*“I order takeout at work. I know takeout is unhealthy (a GDM risk), but I was so busy at work that I didn’t have time to choose other healthy foods.” (G5)*.

#### Attitudes and perceptions of GDM

All pregnant nurses with GDM in the study described GDM as a controllable disease, and understood that adverse effects on the fetus could be reduced if the blood glucose is controlled. Several nurses said that GDM is a widespread condition and they should not worry about adverse pregnancy outcomes. Other nurses were reluctant to talk about GDM and felt guilty and stigmatized for their poor blood glucose control. Some nurses interpreted GDM as a threat to their health and wanted to maintain a healthy lifestyle during pregnancy and the postpartum period.

*“It (diet management of GDM) is excruciating. I always say, after delivery, I want to eat cake and fruit. I try to control myself deliberately at present.” (G5)*.

### Experiences of lifestyle changes

The second theme of this study was the indication by pregnant nurses with GDM that lifestyle changes based on existing medical knowledge could help them cope with the complex glycemic management of GDM. Nevertheless, they described the significant challenges involved in maintaining a healthy lifestyle and the difficulty in balancing their busy clinical work and self-care. Regular blood glucose monitoring was unsatisfactory in terms of time and brought emotional turmoil and self-reproach due to pain and abnormal blood glucose levels. Three sub-themes were identified under this theme.

#### Conflict between diet management and clinical work

We found that pregnant nurses with GDM could appreciate the relationship between the blood glucose results and daily diet and could actively change and adjust their diets. However, many nurses said that that the adjustment to the GDM lifestyle required significant effort especially as they had to balance the proper diet with busy clinical work and shift work. They had to give up dietary management during working hours because strategies to reduce the amount and portion size of meals are difficult to implement. The pregnant nurses with GDM also reported guilt, anxiety, and frustration about the health of the fetus when the lack of dietary management resulted in abnormal blood glucose levels.

*“I’m always busy with clinical work and don’t have time to stop. There’s no way to get much time to eat (sigh of resignation). So, I need to eat quickly and eat filling food every time I eat.” (G8)*.

#### Fatigue from physical labor

In this study, pregnant nurses with GDM reported significant levels of fatigue resulting in physical and emotional labor, shift work, and the intense pace of their daily clinical work, with no extra time for physical activity. At the same time, they also indicated that the physical labor of clinical work was heavy, and that the amount of walking done was more than 10,000 steps per day, which they believed met the energy consumption required for GDM. Exhausted from nursing work, nurses described going home and wanting to lie and rest or sleep. In addition, nurses with specific pregnancy conditions, such as artificial insemination or pregnant women with scars for a short time, were cautious and worried about clinical work and exercise.

*“I am busy at work (as a nurse). I do approximately 15,000 steps per day. I rarely schedule a time to exercise alone anymore. I am tired, mentally and physically.” (G6)*.

#### Blood glucose monitoring and emotional shock

Pregnant nurses with GDM reported understanding the importance of self-monitoring blood glucose as they were caregivers for others, as well as having a clear understanding of the impact of a healthy lifestyle. Some nurses said blood glucose measurement was convenient in hospital work. At the same time, they hoped to get help from colleagues but did not want to increase the burden on colleagues.

Furthermore, all pregnant nurses with GDM reported that their daily emotions, sleep patterns, and lifestyles were affected by the blood glucose-monitoring results. Meanwhile, the nurses also described guilt and remorse when their blood glucose was abnormal, and they were at a loss. We also found that nurses were resistant to the use of insulin. Even though endocrinologists emphasized the safety of the drug’s use, they were still worried and fearful about possible side effects of the drug on fetal health. This corresponds to the Chinese proverb that drugs are seven parts effective and three parts toxic.

*“The doctor advised me to be hospitalized and injected insulin to adjust my blood glucose. I’m scared. But then I got my blood glucose under control with diet and physical activity, and I was so glad.” (G7)*.

*“It’s hard for me to find time to test blood glucose in my clinical work. I occasionally measure my blood glucose and find it extremely high, leaving me devastated and overwhelmed.” (G9)*.

### Social support needs

The third theme of this study was the stated need by pregnant nurses with GDM for social support and the observation that clinical work stress, such as overburdening with work and specific shift systems, conflicted with the lifestyle management required. Nurses have subject to high levels of expectation for humanistic care from leaders. However, most of the time, family and professional support could help them cope with a complex condition. Three sub-themes were identified under this theme.

#### Humanistic care needs

Nurses expressed concern that overcrowded working conditions and the need to deal with emergencies resulted in high levels of mental stress, especially during COVID-19, with little energy left over to pay attention to pregnancy status and blood glucose. At the same time, they were also concerned that the working hours of the shift system, especially the night shift, could harm the health of both themselves and the fetus. Most of the nurses in the study said they received care and help from the nursing managers to reduce their work burden as much as possible. For example, nursing managers would reduce clinical work schedules, reduce night shifts, arrange easy administrative work, and other ways to take humanistic care of nurses with GDM.

*“We must work until 37 weeks before we can take maternity leave, and most jobs involve lifting patients. So, my nursing work is very stressful and busy. I can’t feel like a pregnant woman, and it was hard (crying). I also didn’t take the time to manage my blood glucose.” (G1)*.

*“Working night shifts during pregnancy was tiring, and the disturbed sleep was terrible for my blood glucose. After work, I want to sleep and do nothing. I am worried about my baby’s health, but in this profession, there is no other way (helpless sigh).” (G9)*.

#### Family support needs

The pregnant nurses with GDM agreed that family support helped with pregnancy and blood glucose management and eased their anxiety. They reported that care and encouragement from their partners provided both emotional support and assistance or participation in encouraging blood glucose management behaviors among nurses. Some pregnant nurses with GDM described feeling happy to be recognized for sharing a healthy lifestyle with family members.

*“He (husband) encouraged me to control it (blood glucose).” (G8)*.

*“I share healthy eating ways with my family. They recognize my expertise and eat the same food as I do.” (G6)*.

Some pregnant nurses with GDM expressed that ingrained traditional dietary beliefs affected the relationships between the participants and their mothers-in-law. For example, conventional cultural habits believe that eating rich food (such as high carbohydrate foods and the soup of the day) and more fruit is necessary during pregnancy to promote fetal development. The nurses said they understood the elders’ concerns, but that the recommendations would result in overnourishment. Nurses are knowledgeable about nutrition and can adequately self-care in this area.

*“I don’t like hearing my mother-in-law’s lessons, such as eating more (high-fat) soup, rice, and fruit, which can promote foetal development. I think these perceptions are problematic. However, they are concerned and ask me to do what they want (helpless smile, sigh).” (G7)*.

#### Available professional support

The pregnant nurses with GDM had a different willingness to learn new GDM knowledge. All nurses were self-rated that they had a basic knowledge of GDM, blood glucose control, and its adverse effects on the fetus and themselves. Most pregnant nurses were not willing to participate in or occasionally participate in the GDM courses offered by the hospital. They explained that they were too busy at work and believed that their medical knowledge was sufficient. However, the study found that pregnant nurses were not sufficiently clear about their knowledge of GDM and were not enthusiastic about professional support. Other nurses stated that it was easier to get help from obstetricians in the hospital, which reduced negative emotions about adverse fetal outcomes. In addition, they could share their blood glucose status with colleagues at any time to get encouragement and comfort.

*“I occasionally attend classes at the diabetes day clinic, and also dos online study on GDM. I think that I have adequate knowledge on blood glucose control so this does not need to be learned.” (G7)*.

A few other pregnant nurses with GDM actively acquired knowledge of GDM and had good medical knowledge. These nurses felt reassured that enough professional information could help manage the illness and maintain good mental equilibrium. In addition, two nurses expressed horror because they were well aware of the adverse risks of GDM to the fetus and their own high risk of diabetes.

*“Although I am a nurse, I do not work in endocrinology or obstetrics and thus I don’t have specific knowledge of GDM. I have taken online courses to learn about GDM. This information and understanding eased my anxiety.” (G4)*.

### Health expectations and risk perception

The fourth theme identified in this study was the nurses’ perception of the threat of GDM to their own health and that of the fetus. They stated that blood glucose management was the responsibility of the mother and the health of the fetus was the driver of lifestyle change. In addition, pregnant nurses with GDM had concerns and fears about weight, shame, and future health risks. Two sub-themes were identified under this theme.

#### The responsibilities and expectations of motherhood

Successful behavioral change depends on many factors, and the fetus is the most crucial factor in blood glucose management. The pregnant nurses with GDM expressed responsibility for the health of their babies. We found that pregnant women with GDM had more had an increased knowledge of the adverse pregnancy outcomes that added to their worries concerning their children’s health.

*“When I feel terrible about my diet and lack of exercise, I’m very scared. Because I underwent IVF (In-vitro fertilization) many times. Now I have GDM, which makes me even more worried about the effect on the baby.” (G4)*.

*“I’m a little worried, worried… polyhydramnios, macrosomia, miscarriage, premature delivery. Hypoglycaemia after birth… I’m mainly worried about my baby.” (G9)*.

#### Awareness of self-health and image

Most nurses with GDM said they were mainly worried about the baby’s health not themselves. They generally believed that the blood glucose levels would naturally return to normal after childbirth. In addition, some nurses with GDM were well aware that a history of GDM carries a high risk of developing T2DM and felt afraid of financial burden of diabetes. Some GDM nurses also expressed dissatisfaction and anxiety about the impact of excessive weight gain during pregnancy on their self-image, and there was weight shame.

*“I’m afraid I will get diabetes. People with diabetes have daily blood glucose tests and insulin injections, and there is no cure.” (G5)*.

*“I’m worried I’ll still be this fat postpartum.” (G2)*.

## Discussion

This study explored the experiences and feelings of pregnant nurses in China after a diagnosis of GDM. The results showed that pregnant nurses with GDM have medical knowledge to help them understand the disease and blood glucose management. Most nurses said they understood the importance of a healthy lifestyle for blood glucose but found balancing busy clinical work with blood glucose management challenging. In addition, as nurses take care of others, they spend most of their time taking care of patients, so they often lack the ability or motivation to care for themselves. At the same time, nurses felt helpless about the adverse effects of work overload, especially the demanding working conditions, long working hours, and shift work, on managing GDM and the fetus. Pregnant nurses with GDM expressed their desire to receive more care and special care from nursing managers to minimize adverse pregnancy outcomes.

Pregnant nurses experienced strong feelings of shock, fear, sadness, guilt, and anxiety when diagnosed with GDM. In a review study, women were found to suffer from emotional disorders [15], and some women experienced excessive fear, helplessness, stigma, and self-blame due to information gaps after a GDM diagnosis [26]. In this study, patients with medical knowledge of GDM were more inclined to evaluate and analyze their physical condition based on diagnostic criteria and blood glucose levels. Compared to pregnant women with less knowledge of GDM, women with a medical background had a shorter interval between the shock of diagnosis and emotional plateau and had confidence in proper glycaemic control.

Pregnant nurses with GDM face many challenges in learning to adjust their diet and exercise habits. Social and cultural backgrounds also influence eating habits. For example, rice and pasta are the main meals in China, so it is difficult to adjust the intake of staple foods [27]. However, it was found to be often difficult for nurses with GDM to balance dietary management and demanding clinical work during pregnancy. Failure to manage blood glucose levels adequately can significantly increase the anxiety and stress during pregnancy [15, 28].

Physical activity is another crucial factor in controlling blood glucose levels. The nurses said they took over 10,000 steps daily during their clinical work. Most nurses with GDM stated that they were physically and mentally exhausted due to the high work load and long-term mental stress and were not able to arrange extra time for physical activities [5]. Moreover, all pregnant nurses with GDM recognized the importance and convenience of monitoring blood glucose. However, only a few nurses with GDM conducted regular blood glucose monitoring, and the busy work pace was an obstacle to blood glucose monitoring. However, women with GDM found that abnormal blood glucose readings increased their feelings of guilt and self-blame [29]. Both nurses and women in general with GDM were found to poor compliance with routine monitoring, which is significant for neonatal outcomes [30].

In the study, all nurses with GDM used lifestyle changes to control blood glucose, and expressed rejection, worry, and fear about the use of insulin. The pros, cons, and importance of drug therapy and blood glucose management should be emphasized and refined in GDM health guidance [15, 31]. Furthermore, nurses with GDM experience pregnancy stress and emotional stress in addition to their demanding clinical work and shift-work loads, which can further worsen their islet function [12]. Pregnant nurses experience various difficulties due to the physical and mental changes occurring during pregnancy, and the demanding clinical working environment requires greater humanistic care [3]. Support from partners and other family members can ease a woman’s anxiety during pregnancy and increase her confidence and enthusiasm in managing GDM [32].

Studies have shown that educational courses about GDM were considered feasible for learning about glycemic management and risk perception [15, 33]. While the nurses with GDM lacked precise relevant knowledge, the study found that they took the initiative to acquire knowledge about GDM when needed and could easily understand it. Poor management of GDM in women in general is associated with a lack of adequate and understandable health education [34]. In addition, Dayyani et al. emphasized that a false sense of security should not arise by informing the offspring of the possibility of blood glucose recovery after birth [26].

We found that women’s focus and concern for fetal health is the key promoter of glucose management, which agrees with many previous studies [35, 36]. However, some nurses with GDM described fear and stigma associated with the possibility of adverse risk outcomes, and expressed guilt or anxiety about poor glycemic management behavior [37, 38]. The stigma associated with body image resulting from excess weight gain during pregnancy and obesity is also widely recognized [39]. During the interview, we learned that most women with GDM are aware of the risk of type 2 diabetes, but the risk awareness is related to education level and medical background. In general, health awareness and risk consciousness are related to the level of knowledge of GDM [15].

Pregnancy and GDM experienced by nurses, who are core providers of health services, should not be seen as problems that individuals suffer in isolation [18]. Pregnant nurses with GDM should receive care from nursing managers, active improvements to their working environment, and encouragement for self-care and the promotion of their well-being experience and the ability of nurses with GDM to self-care, we can lay a foundation for providing accurate and personalized medical services and management measures.

### Study limitations

Our study had several limitations. Although the researchers and participants were in a quiet and undisturbed office during the interview, it was inevitable that participants would be interrupted by calls they needed to answer, which may have impacted their thinking during the interview. As with all qualitative studies, the intent of these results is not to generalize, as they are specific to the participants’ experiences, which were those of a relatively small number of nurses with GDM. The physical and mental burdens of pregnant nurses with GDM increased during COVID-19 with the additional tasks of nucleic acid testing and high levels of patient management. The findings provide an understanding of the experiences of pregnant nurses during that disastrous era.

## Conclusions

The study’s results provide information on nurses’ emotional responses, disease experiences, and self-care abilities after being diagnosed with GDM. In addition, many factors, such as unique occupational environment, demanding and pressured work often leading to overwork, long hours and shift work, family status, and education level may lead to difficulties in the management of blood glucose levels in nurses with GDM. Based on these findings, we strongly encourage nurses and healthcare managers to pay more attention to pregnant nurses with GDM and develop personalized medical care and work arrangements. These measures could improve self-care and well-being in nurses with GDM and promote the health of both the nurses and their offspring.

### Electronic supplementary material

Below is the link to the electronic supplementary material.

Supplementary Material 1

## Data Availability

All data generated or analyzed during this study are included in this published article.
